# Pomegranate Juice and Extract Consumption Increases the Resistance to UVB-induced Erythema and Changes the Skin Microbiome in Healthy Women: a Randomized Controlled Trial

**DOI:** 10.1038/s41598-019-50926-2

**Published:** 2019-10-10

**Authors:** Susanne M. Henning, Jieping Yang, Ru-Po Lee, Jianjun Huang, Mark Hsu, Gail Thames, Irene Gilbuena, Jianfeng Long, Yunhui Xu, Esther HaeIn Park, Chi-Hong Tseng, Jenny Kim, David Heber, Zhaoping Li

**Affiliations:** 1 Center for Human Nutrition, David Geffen School of Medicine, Department of Medicine, Los Angeles, CA 90095 USA; 2 Department of Clinical Nutrition, 2nd Xiangya Hospital of Central South University, Changsha, Hunan 410011 China; 3 Department of Statistics Core, David Geffen School of Medicine, University of California Los Angeles, Los Angeles, CA 90095 USA; 4 Division of Dermatology, David Geffen School of Medicine, University of California Los Angeles, Los Angeles, CA 90095 USA

**Keywords:** Clinical microbiology, Outcomes research

## Abstract

*In vitro* and animal studies have demonstrated that topical application and oral consumption of pomegranate reduces UVB-induced skin damage. We therefore investigated if oral pomegranate consumption will reduce photodamage from UVB irradiation and alter the composition of the skin microbiota in a randomized controlled, parallel, three-arm, open label study. Seventy-four female participants (30–45 years) with Fitzpatrick skin type II-IV were randomly assigned (1:1:1) to 1000 mg of pomegranate extract (PomX), 8 oz of pomegranate juice (PomJ) or placebo for 12 weeks. Minimal erythema dose (MED) and melanin index were determined using a cutometer (mexameter probe). Skin microbiota was determined using 16S rRNA sequencing. The MED was significantly increased in the PomX and PomJ group compared to placebo. There was no significant difference on phylum, but on family and genus level bacterial composition of skin samples collected at baseline and after 12 week intervention showed significant differences between PomJ, PomX and placebo. Members of the Methylobacteriaceae family contain pigments absorbing UV irradiation and might contribute to UVB skin protection. However, we were not able to establish a direct correlation between increased MED and bacterial abundance. In summary daily oral pomegranate consumption may lead to enhanced protection from UV photodamage.

## Introduction

Exposure of human skin to UV radiation is a major factor for skin pathologies including erythema, inflammation, degenerative age-related changes and cancer^1^. UV radiation is mostly composed of UVA (315–400 nm) and UVB (290–320 nm). Overexposure of the skin to UVA and to a lesser extent to UVB leads to oxidative stress that increases the generation of reactive oxygen species (ROS) causing lipid peroxidation of cell membranes, DNA and protein damage to tissue, inflammation and keratinocyte apoptosis^2–4^. ROS also trigger the expression of matrix metalloproteinases (MMP), that degrade extracellular matrix such as collagen maintaining cell and skin integrity^3^.

Pomegranate fruits have been used for centuries in ancient cultures for its medicinal purposes^5^. The health benefit of pomegranate has been attributed to the content of hydrolysable tannins (ellagitannins) including punicalagins and ellagic acid (EA) as well as anthocyanins and other polyphenols found in pomegranate extract and juice^6^. Although POM ellagitannins are highly bioactive *in vitro*, they are not absorbed intact in the small intestine and undergo partial hydrolysis to form EA^7^. Ellagitannins and EA remaining in the large intestine are further metabolized to urolithins A –D by the microbiota in the large intestine and are absorbed into the blood stream^8–10^. Therefore, pomegranate properties could be mediated by the metabolites produced in the intestine in addition to the original phenolic compounds present in the food matrix. Due to the sugar content of PomJ, which might be of health concern, we included both PomJ and PomX intervention in this investigation.

Several *in vitro* and animal studies provide evidence that either topical application or oral consumption of pomegranate juice (PomJ) or extract (PomX) or EA reduce damage from UVB irradiation^11–17^. One human study demonstrated that oral consumption of an EA rich pomegranate extract in healthy women was associated with a protective effect on slight sunburn caused by UV irradiation even at a low dose resulting in a decrease in pigmentation^17^. *In vitro* antioxidant and anti-inflammatory activity of ellagitannins and EA have been widely demonstrated^18–20^, while oral ingestion of pomegranate in humans has been less investigated. Some human studies provide mechanistic evidence that pomegranate ingestion leads to an increase in antioxidant and anti-inflammatory activity^9,21^.

Inflammation caused by UV radiation activates various matrix-degrading matrix metalloproteases (MMPs), which leads to collagen degradation and cellular apoptosis. MMP-1, especially, was the main endogenous factor that degraded dermal collagen in the process of human skin senility^22^.

Previously published findings from our laboratory demonstrated that pomegranate extract inhibited *in vitro* growth of bacteria involved in the pathogenesis of acne including *Propionibacterium acnes*, *Propionibacterium granulosum*, *Staphylococcus aureus* and *Staphylococcus epidermidis*^23^. Although the concentration of circulating Pom ellagitannin metabolites is very low, there is a possibility that Pom ellagitannin metabolites in the skin might alter the composition of the skin microbiota^8^. In addition, pigment forming bacteria can contribute to skin UV protection^24^. Therefore, it was our hypothesis that pomegranate consumption might alter the skin microbiota contributing to protection from UVB.

The objective of the current study was to determine whether pomegranate extract (PomX) or pomegranate juice (PomJ) can decrease UVB-induced skin photoaging, alter inflammatory markers and the skin microbiota.

## Results

### Characteristics of participants

Seventy-four participants completed the study. There was no statistically significant difference at baseline for average age, height, weight, BMI, race, ethnicity and skin type **(**Table 1). Although there were more Asian women in the PomJ group compared to PomX and placebo, there was no difference in skin type distribution among the three groups (Table 1).

**Table 1:** Baseline demographics of study participants (n = 24–25).

	Pom Juice (n = 24)	Pom extract (n = 25)	Placebo (n = 25)	P value
Age (years)	35.1 ± 4.3	35.9 ± 4.1	37.9 ± 4.2	0.063
Height (inches)	63.5 ± 2.8	63.9 ± 2.6	63.8 ± 2.5	0.843
Weight (lbs)	153 ± 30.8	158.7 ± 34.5	170.2 ± 40.7	0.253
BMI	26.6 ± 5.0	27.1 ± 5.1	29.9 ± 6.7	0.092
Female	24 (100)	25 (100)	25 (100)	
Race:White	14 (58)	20 (80)	14 (56)	0.284
Black	0	1 (4)	1 (4)	
Asian	9 (38)	3 (12)	3 (12)	
Bi-racial	1 (4)	1 (4)	2 (8)	
Ethnicity:Hispanic	9 (37)	16 (64)	10 (40)	0.119
Non-Hispanic	15 (63)	9 (36)	15 (60)	
Skin Type				0.552
II	5 (20.8)	2 (8.0)	3 (12.0)	
III	6 (25.0)	9 (36.0)	11 (44.0)	
IV	13 (54.2)	14 (56.0)	11 (44.0)	
Melanin index (RU)	264.5 ± 212.6	242.7 ± 91.1	202.2 ± 75.4	0.59
UAG producer	16 (67)	19 (83)	n/a	
UAG non-producer	8 (33)	4 (17)#	n/a	

Data are mean ± SD. Numbers in parenthesis are percent. #two participants did not produce urolithin A glucuronide (UAG) or dimethylellagic acid glucuronide (DMEAG), relative unit RU.

### Urolithin formation

In the intestine pomegranate ellagitannins are broken down to ellagic acid (EA), which can be absorbed and converted to methylellagic acid glucuronide (DMEAG). Urinary DMEAG can be used to determine compliance. EA remaining in the intestine is further metabolized by bacteria to urolithin A (UA). After absorption UA circulates in form of UA glucuronide (UAG). In the PomJ group, 16 participants (67%) showed UAG in urine and 8 (33%) were UA non-producers, while in the PomX group 19 participants (83%) formed UA and 4 (17%) were UA non-producers. In the PomX group two participants had neither UAG nor DMEAG in urine, which may indicate that on the day prior to the blood collection the participants did not consume the pomegranate product. They were excluded from the statistical analyses of the skin measurements. There was no significant difference in urine UAG and DMEAG between individuals consuming PomJ or PomX (Table 2).

**Table 2:** Minimal erythema dose and urine urolithin A glucuronide and dimethylellagic acid glucuronide in UA producers and non producers consuming PomJ, PomX or placebo.

	Pom Juice UA producer (n = 16)	Pom Juice nonUA producer (n = 8)	Pom extract UA producer (n = 19)	Pom extract nonUA producer (n = 4)	Placebo (n = 25)
Urine UAG (ng/mL)	3147 ± 1861	0	4068 ± 2049	0	n/a
Urine DMEAG (ng/mL)	163 ± 147	199 ± 118	210 ± 155	200 ± 101	n/a
MED BL (mJ/cm^2^)	385.7 ± 97.7	379.3 ± 100.1	409.2 ± 80.6	347.5 ± 111.4	384.2 ± 105.6
MED F (mJ/cm^2^)	417.5 ± 126.6	420.0 ± 87.6	440.0 ± 88.1*	373.8 ± 41.1	367.2 ± 90.2

Data are mean ± SD, n = 24–25. ANCOVA model was used to compare outcomes adjusted for baseline value between placebo and Pom extract or placebo and Pom Juice groups; *p < 0.05. Urolithin A glucuronide (UAG); dimethylellagic acid glucuronide (DMEAG); minimal erythema dose (MED); baseline BL; final (F).

### Effects of POM extract consumption on minimal erythema dose and melanin index

To evaluate the minimal erythema dose (MED) the inner arm skin was exposed to increasing dose and exposure time to determine the minimal UVB dose that induces erythema. In women consuming PomJ (p = 0.038) or PomX (p = 0.011) for 12 weeks the MED was increased significantly when analyzing the difference of baseline to final erythema dose compared to placebo group. In the PomX group UA producers experienced a significant increase in MED, while in UA non-producers MED was not increased significantly (Table 2). In the PomJ group, there was no difference between UA producers and non-producers in regards to MED. The time of exposure showed a trend to increase in both groups compared to the placebo group (p = 0.083 for PomJ and p = 0.089 for PomX).

Upon exposure to UVB irradiation epidermal melanocytes produce melanin that is transferred to neighboring keratinocytes protecting the cells from UV radiation damage^25^. However, hyperpigmentation will lead to premature photoaging and senescence in melanocytes^25^. Melanin formation also can be used to evaluate the effect of UVB exposure. In the present study in women consuming PomJ and PomX melanin concentration was decreased but did not reach significance (Table 3).

**Table 3:** UVB-induced minimal erythema dose, time of exposure and skin characteristics determined before and after PomX, PomJ and placebo intervention.

	Pom Juice (n = 24)	Pom extract (n = 25)	Placebo (n = 25)
Compliance F (%)	96.3 ± 5.1	97.5 ± 3.9	98.9 ± 2.1
MED BL (mJ/cm^2^)	383.6 ± 95.8	396.8 ± 83.6	384.2 ± 105.6
MED F (mJ/cm^2^)	418.3 ± 113.1*	429.6 ± 81.8*	367.2 ± 90.2
Time BL (sec)	175.5 ± 42.9	193.7 ± 46.7	185.6 ± 52.5
Time F (sec)	199.1 ± 58.6	203.6 ± 49.0	177.2 ± 43.6
Melanin index BL (RU)	264.5 ± 212.6	242.7 ± 91.1	202.2 ± 75.4
Melanin index F (RU)	195.0 ± 63.9	219.6 ± 61.0	198.9 ± 67.4
Sebum BL (µg/cm^2^)	17.0 ± 19.7	14.8 ± 17.5	22.1 ± 36.8
Sebum F (µg/cm^2^)	15.3 ± 24.4	15.2 ± 18.2	26.9 ± 37.7
Hydration BL (RU)	41.8 ± 14.4	36.4 ± 12.6	41.9 ± 14.5
Hydration F (RU)	38.7 ± 9.8	41.4 ± 11.4	40.9 ± 10.8

Data are mean ± SD, n = 24–25. BL = baseline, F = final, RU = relative unit. ANCOVA model was used to compare outcomes adjusted for baseline value. *p < 0.05. Minimal erythema dose (MED); baseline BL; final (F).

### Effects of POM extract consumption on the skin microbiota

**Figure 1:**
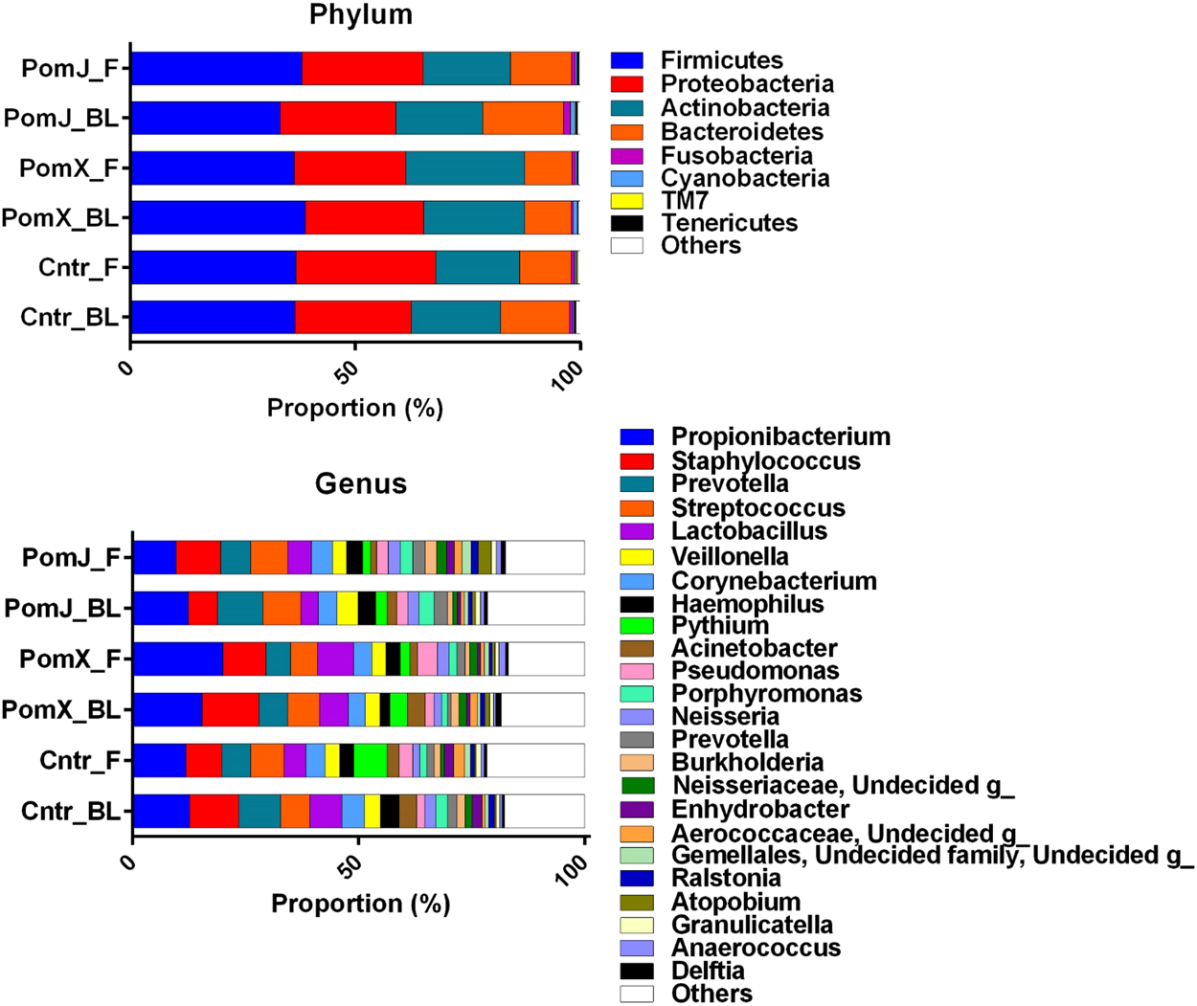
Relative abundance of skin microbiota before and after pomegranate and placebo intervention. Stacked column bar graphs depict the average relative abundance and distribution of the most abundant resolved taxa at the phylum (**A**) and genus (**B**) level before (BL) and after (12 week, F) PomX, PomJ and placebo control (Cntr) intervention.

Pomegranate ellagitannins and EA have anti-bacterial activity *in vitro* and topical application of a Pom extract ointment has been shown to decrease the growth of *Propionibacterium acnes* and reduce edema in Wistar rat ears^23,26,27^. In the present study we determined whether oral consumption of PomJ or PomX altered the microbiota on the skin surface. Microbiota analysis showed the following composition of the skin microbiota on the phylum level: 36–38% Firmicutes, 25–31% Proteobacteria, 18–30% Actinobateria, 9–17% Bacteroidetes, 0.2–1.9% Fusobacteria, 0.2–1.1% Cyanobacteria, 0.003–0.4% Tenericutes and 0.2–1% others (Fig. 1A). Intergroup analysis showed no significant difference in change of phylum composition of skin samples collected at baseline and after 12 week intervention from women consuming PomJ compared to placebo and PomX and placebo.

Intergroup analysis comparing the change in relative abundance of skin bacteria from baseline to week 12 between each intervention group (placebo, PomX and PomJ) showed multiple significant changes, which were not always in the same direction for PomX and PomJ intervention. We focused our data analysis on bacteria with relative abundance that was significantly different between the pomegranate and placebo groups.

**Figure 2:**
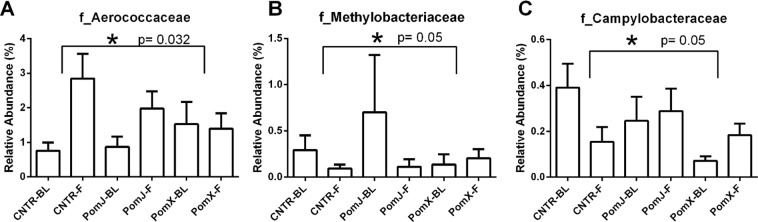
Relative abundance of skin bacteria at the family level that were significantly different comparing PomX to placebo control (Cntr) and PomJ to placebo control groups (change from BL to 12 weeks [F]). Data are mean ± SD, n = 24–25. Non-parametric Kruskal–Wallis with Mann-Whitney test was used. Bonferroni correction was used to correct the probability for multiple comparisons.

**Figure 3:**
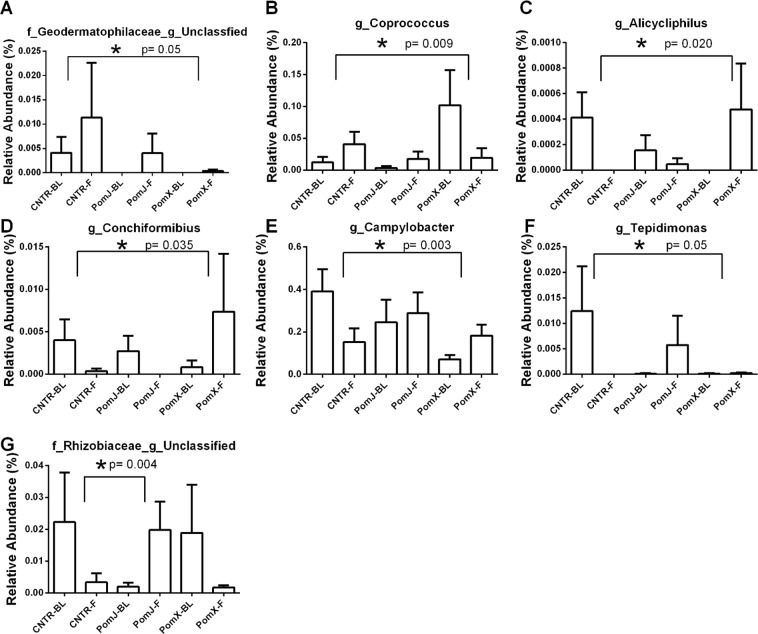
Relative abundance of skin bacteria at the genus level that were significantly different comparing PomX to placebo control (Cntr) and PomJ to placebo control groups (change from BL to 12 weeks [F]). Data are mean ± SD, n = 24–25. Non-parametric Kruskal–Wallis with Mann-Whitney test was used. Bonferroni correction was used to correct the probability for multiple comparisons.

On the family level bacteria from the family *Aerococcaceae*, *Methylobacteriaceae* and *Campylobacteraceae* were altered significantly with PomX consumption compared to placebo (Fig. 2). Intergroup analysis comparing the change of bacterial abundance from week 12 to baseline showed that *Campylobacteraceae* (Proteobacteria) was increased and *Methylobacteriaceae* not changed in the PomX group, while a decrease was observed in the placebo group (Fig. 2). The relative abundance of Aerococcaceae was increased in the placebo group, but there was no change in the PomX group (Fig. 2). The changes for PomJ compared to placebo group were not significant. On the genus level the most frequently occurring bacteria included *Propionibacterium* (Actinobacteria) > *Staphylococcus* (Firmicutes) > *Prevotella* (Bacteroidetes) > *Streptococcus* (Firmicutes) > *Lactobacillus* (Firmicutes) > *Corynebacterium* (Actinobacter) > *Veillonella* (Firmicutes) > *Haemophilus* (Proteobacteria) > *Acinetobacter* (Proteobacteria) (Fig. 1B). On the genus level comparing the PomX to placebo group, five genera were changed in opposite direction compared to the placebo group: Coprococcus (Firmicutes) was decreased, Alicycliphilus, Conchiformibius and Campylobacter (Proteobacteria) were increased and Geodermatophilaceae_unclassified was not changed comparing week 12 to baseline (Fig. 3). Comparing PomJ group to placebo we determined that the unclassified genus from the family Rhizobiaceae was changed significantly (Fig. 3).

**Figure 4:**
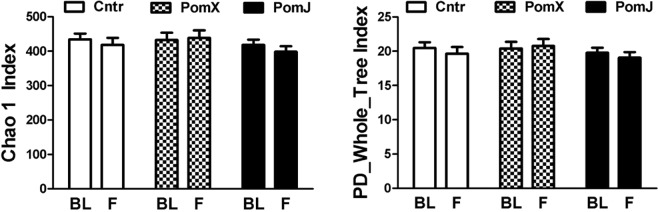
Diversity analyses of skin microbiota before (BL) and after (F) PomX, PomJ and placebo control (Cntr) intervention. Alpha diversity using Chao1 index (**A**) and Whole tree index (**B**) was evaluated using QIIME software package. Data are means ± SD (n = 24–25).

**Figure 5:**
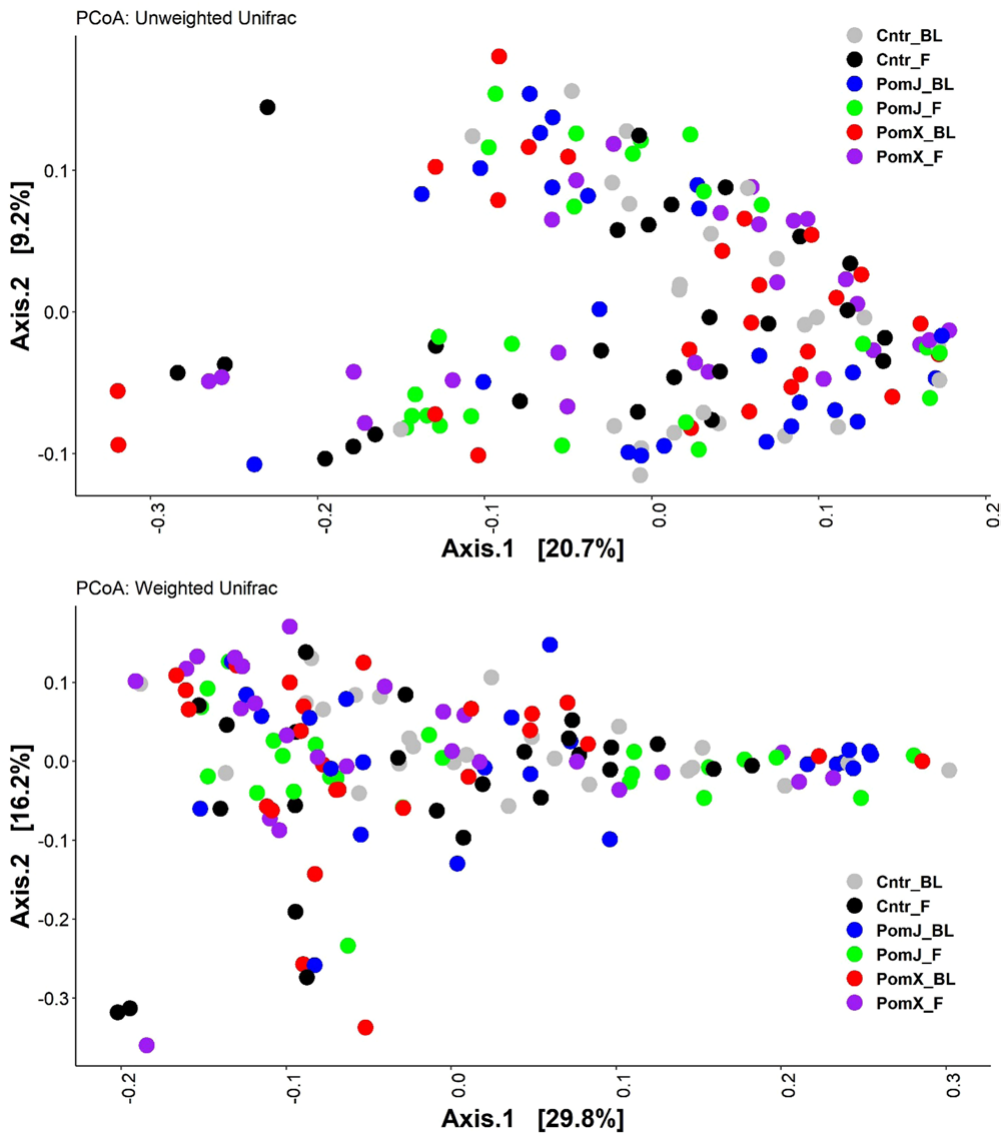
Beta-diversity analyses of skin microbiota before (BL) and after (F) PomX, PomJ and placebo control (Cntr) intervention. Unweighted (**A**) and weighted (**B**) UniFrac PCoA plots were created using QIIME software package.

Pomegranate consumption had no effect on alpha diversity and no clusters were observed in the principal coordinate analysis of weighted and unweighted beta-diversity (Figs 4 and 5).

## Discussion

The main findings observed in the present study were a significant increase in minimal UVB dose to induce erythema in women consuming 1000 mg of PomX or 8 oz of PomJ daily for 12 weeks. The minimal erythema dose is determined by the lowest UVB dose and time of exposure to induce skin erythema. In the present study we also observed a non-significant trend to increase in time of UVB exposure (Pom X p = 0.08 and PomJ p = 0.088) and a non-significant decrease in melanin formation (Pom J p = 0.16). Together the results demonstrate that pomegranate consumption may lead to increased protection to UVB-induced damage to skin.

Possibly the MED might have been increased due to systemic pomegranate metabolites such as dimethylellagic acid glucuronide or urolithin A glucuronide circulating in the blood stream. Since we found that in the Pom X group urolithin A producers showed a significant increase in MED compared to non-producers, we suggest that circulating urolithin A glucuronide is involved in the UVB protection.

In previous *in vitro* and animal studies the topical application of pomegranate and EA improved the resistance of skin to UVB exposure^12^. For example, the study by Bae JY *et al*. demonstrated that topical application of EA reduced collagen breakdown by inhibiting matrix metalloproteinase (MMP) activity and inflammation in UVB-irradiated human skin cells and hairless mice^12^. In addition, animal studies also showed that oral consumption of pomegranate and EA prevented UVB-induced skin damage^4,28,29^. For example oral consumption of PomJ concentrated powder in hairless mice resulted in reduction of UVB-induced skin wrinkles through increased skin water content, collagen type I and hyaluronan content^4^. Other processes reported in the literature, contributing to pomegranate’s UVB photoprotection of the skin include oxidation, inflammation, melanin formation, apoptosis of keratinocytes, activity of matrix metalloproteinases (MMPs), collagen and elastin formation and were identified in *in vitro* studies in human skin fibroblasts, keratinocytes or reconstituted skin^13,14,30^.

Previously, one human study has been performed testing the effect of oral consumption of a high EA pomegranate extract for 4 weeks on pigmentation in the skin caused by UV irradiation and showed a trend, but no significant change in erythema^17^. Possibly, in this study by Kasai *et al*. 4 weeks of pomegranate consumption was not long enough to see a significant change. Another human study demonstrated that oral consumption of a phytonutrient blend containing omega-3 fatty acids, resveratrol, quercetin, and other polyphenols led to protection against UVR-induced skin damage^31^.

Skin exposed to UVB irradiation develops symptoms of a mild sunburn associated with inflammatory response and characterized clinically by redness, which is mediated by increased dermal vascular permeability, vasodilation, edema and inflammatory cell infiltration^32^. In the current study we did not observe a change in skin hydration at the end of pomegranate consumption for 12 weeks.

A likely target of Pom metabolites is melanin formation^28,30^. We observed a trend to decrease of melanin formation comparing melanin concentration before and after pomegranate consumption. It has been previously demonstrated that EA and pomegranate concentrate inhibited tyrosinase activity, the enzyme necessary for melanin formation^28^. Administration of pomegranate extract inhibited pigmentation, in a dose-dependent manner, on the skin of brownish guinea pigs receiving UV irradiation, where the number of melanocytes in the epidermis was decreased in a dose-dependent manner^28^. These results suggest that EA orally administered is absorbed into the body and the EA and/or its metabolites inhibit proliferation of melanocytes in the skin, resulting in inhibited synthesis of melanin by tyrosinase in melanocytes.

Other potential mechanisms of protection from photoaging are through anti-apoptotic effects, inhibition of MMP activity, inhibition and extracellular matrix (ECM) (COL1 and hyaluronan) synthesis- related moisturizing, anti-inflammatory and antioxidative effects, which have been observed in healthy female SKH-1 hairless mice receiving oral gavage of 100–400 mg/kg body weight of a dried PomJ concentrated powder^4^. In the present study, we did not find any changes in gene expression of markers of inflammation (Smad3, Tgfβ) and photoaging (MMP1, β-integrin, stratifin and IGF1R) (data not included). Samples were collected by tape stripping of skin locations not exposed to UVB since UVB exposed skin was irritated and tape stripping could cause considerable skin irritations. However, since no photo challenge was induced to the skin prior to tape stripping we did not observe any protection with pomegranate consumption.

Human skin microbiota composition depends on the location of the skin on the body^33^. Skin swabbing samples for microbiota analysis were collected at the inner elbow (antecubital fossa), which is considered a moist but not oily habitat. This region is usually enriched for *Corynebacteria* species and *Staphylococci* species^34^. In healthy individuals, the most common skin bacteria are categorized into four different phyla: Actinobacteria (most dominated by *Propionibacterium spp*., and *Corynebacterium spp*.), Firmicutes (major genus is *Staphyloccocus spp*.), Proteobacteria and Bacteroidetes^33,35^. Previous studies have demonstrated that consumption of pomegranate will affect the intestinal microbiota^36^ and *in vitro* studies demonstrated the antibacterial effect of pomegranate on bacteria commonly found on skin, such as *Propionibacterium* and *Staphylococcus*^23^. However, the present study is first to demonstrate that oral consumption of pomegranate altered the skin microbiota. Skin swipes for the microbiota assessment were performed in skin areas of the inner arm, which were not exposed to photoaging. No changes on the phylum level were observed. On the family and genus level several bacteria with minor abundance were changed when comparing PomX and PomJ to placebo. Bacteria in the family Methylobacteriaceae (phylum Proteobacteria) have been found to form UVA-absorbing compounds and are frequently found on plant’s phyllosphere (above ground), exposed to harmful UV irradiation^24^. The proportion of Methylobacteriaceae was decreased in the placebo and PomJ groups and not changed in the PomX group comparing 12 weeks to baseline. The abundance of Methylobacteriaceae was very low and little is known about pigments in other bacteria. Bacterial pigments, however, can potentially contribute to UV skin protection. Other bacteria with altered abundance such as *Campylobacter* (Proteobacteria) and *Coprococcus* (Firmicutes) are not commonly found on skin. Our intergroup evaluation of bacteria abundance showed different effects of PomX and PomJ consumption on the skin microbiota. However, both extract and juice intervention resulted in an increase in UVB protection. Possibly the skin microbiota does not contribute to the UVB protection or since the microbiota functions in a network of many bacteria possibly changes in individual bacteria may not affect the microbiota function.

The evaluation of the skin microbiota was performed in healthy skin at a site without UVB exposure. Therefore, we do not know if the observed changes in the skin microbiota contributed to the increase in UVB protection.

One shortcoming of this study was that it is not possible to sample skin sites shortly after UV exposure. In future studies we will collect skin samples near the site of UV exposure to evaluate the mechanism of skin UVB protection from UVB-induced photodamage and to determine if the skin microbiota contributes to UVB protection.

In summary, PomX and PomJ consumption resulted in an increase in skin protection to UVB exposure as shown by an increase in minimal erythema dose and a trend to decrease melanin formation, indicating an enhancement of UVB protection. Our mechanistic studies did not provide insights into specific targets of the pomegranate induced skin protection mostly because skin and microbiota samples were collected at non-UVB exposed sites. In future studies, we will collect samples near exposed sites to test if changes in gene expression and composition of the microbiota contribute to the UVB protection by pomegranate consumption.

## Methods

This study was a randomized controlled, parallel, three arm open label study completed at the Center for Human Nutrition, University of California Los Angeles, California, USA. The study was carried out in accordance with the guidelines of the Human Subjects Protection Committee of the University of California, Los Angeles. The clinical protocol was approved by the internal review board (IRB) of the University of California, Los Angeles. All subjects gave written informed consent before the study began. The study was registered in ClinicalTrials.gov under the following identifier: NCT02258776 on 10/07/2014.

### Study participants

Seventy-seven healthy women were enrolled. Seventy-four women completed the 12-week pomegranate intervention study. Three participants dropped out related to pregnancy (one participant in the PomX group and one in the PomJ group) and related to moving out of state (one particiant in the the PomJ group). Their data was not included in the statistical evaluation. No adverse effects were reported. Inclusion criteria were 30–40 years of age, female, be in good health. Exclusion criteria were: no use of topical antibiotic or topical steroid on the face, scalp, neck, arms, forearms or hands in the previous 7 days or with any skin condition in the target area, no skin irritations, dry skin or rash and no intake of antibiotics. Throughout the study participants were instructed not to consume pomegranate products, walnuts, or polyphenol-rich fruits (strawberry, raspberry, etc.).

### Study design

Participants were randomized to consuming either pomegranate juice (PomJ 8 oz)(n = 24), pomegranate extract capsules (PomX 1000 mg) (n = 25), or placebo (n = 25) capsules for 12 weeks. The random permuted block design was implemented to carry out the randomization using our standard random number program. Block size was 4 or 6 carried out in a random way. Subjects were instructed to take a daily dose of 1000 mg of the pomegranate extract (POMx®, POM Wonderful, Inc., Los Angeles), which delivers pomegranate polyphenols in an amount equivalent to about 8 oz of pomegranate juice. PomX was developed to be used as a dietary supplement and has Generally Recognized as Safe status^37^. Pomegranate placebo capsules contained inactive excipients (dextran). PomJ contained 100 mg of punicalagin A/B and 23 mg ellagic acid in 240 ml of juice, and PomX contained 100 mg punicalagin A/B and 44 mg ellagic acid in 1000 mg extract, as determined by HPLC^38^.

After subjects signed the informed consent form at baseline, weight and height were measured and skin type was evaluated. At baseline prior to taking pomegranate and at the end of 12 weeks of pomegranate consumption, evaluation of UVB-induced erythema, microbiota, gene expression of epithelial cells and blood collection was performed as described below.

### Study outcomes

The primary outcome of this intervention study was the quantification of the minimal erythema dose (MED) of skin response to UVB exposure and the measure of melanin index in skin. For the secondary outcome changes in the skin microbiota were determined. Both outcomes were determined before (baseline) and after (12 weeks) of PomJ, PomX or placebo consumption.

### Minimal erythema dose, melanin index, hydration and sebum evaluation

We determined the lowest dose of UVB radiation capable of inducing erythema (minimal erythema dose [MED]). The MED was determined for each subject before (week 0) and after (week 12) the intervention. Prior to testing, the skin type was evaluated based on the Fitzpatrick Skin Type scale^39^. Participants with Fitzpatrick skin type 2–4 were included in the study. Background erythema (T0) was measured in all test areas before treatment using a mexameter probe attached to Cutometer dual MPA 580 (Courage&Khazaka electronic GmbH, Koeln, Germany). Skin UVB dose and treatment time were determined based on overall skin type classification. Using the dosing guideline for NB-UVB and the National Biological UVB mJ chart, we determined the sequential exposure times for each skin patch. A sleeve with 6 cut out patches was placed on the subjects arm. Using the Dermalight 90 handheld device (National Biological, Beachwood, OH) the test area on the inner arm of subjects was irradiated with a defined dose of narrow band ultraviolet B (NB-UVB) light delivered by the UV radiation between 270 and 400 nm, peaking at 310 nm was delivered from a fluorescent UV-B lamp (Philips TL20 W/12). Depending on skin type, a dose range of 220–550 mJ/cm^2^ for a time of 100–290 seconds was used. This dose range is typically used for treatment of skin conditions like psoriasis and vitiligo. To evaluate MED the subject returned 24 hours later to determine which skin patch showed minimal erythema (pink color). Photographs were taken before irradiation and after 24 hours. The lowest dose and time of the occurrence of pink were determined and used as MED. Melanin index, hydration and sebum on skin surface were evaluated at baseline (prior to Pom intake) and after 12 week intervention using the mexameter MA18, corneometer CM825 and sebumeter SM815 probes attached to the Cutometer. Sebumeter SM 815 uses the difference of light intensity through a plastic strip to indicate the amount of absorbed sebum. The sebum level is expressed in *μ*g/cm^2^ ^40^. Corneometer CM 825 uses the high dielectric constant of water for analyzing the water-related changes in the electrical capacitance of the skin. It displays hydration measurements in system-specific arbitrary units^40^. A melanin index is calculated by Mexameter MX 18 from the strength of the absorbed and the reflected light at, respectively, 660 and 880 nm. An erythema index is processed similarly at, respectively, 568 and 660 nm^40^.

### Skin surface microbiota collection

Skin sampling using the wet swab method was performed at baseline and 12 weeks. Samples were collected as described by the Human Microbiome Project^41^. A sterile 4 cm square template was placed on the inner arm to mark the sampling area. The collection swab (CatchAll®Sample Collection Swab (Epicenter, Illumina, Madison, WI) was moistened with buffer (50 mM Tris buffer [pH 7.6], 1 mM EDTA [pH 8.0], and 0.5% Tween-20) and the area within the template was swabbed for 30 s rubbing the swab back and forth about 50 times applying firm pressure. The swabs were placed into bead solution for DNA extraction using DNeasy Powerlyzer microbial kit (Qiagen, Valencia, CA) and vortexed for 30 sec. The quality of the extracted DNA was confirmed using the Nanodrop 1000 (Thermo Fisher Scientific, Wilmington, DE).

### Skin microbiological analyses

#### MiSeq sequencing

Microbial sequencing of the V1 to V3 region of 16 S bacterial rDNA was performed using primer pair 27 F (AGA GTT TGA TCC TGG CTC AG) and 534 R (ATT ACC GCG GCT GCT GG)^42^. 30 cycle PCR using the HotStarTaq Plus Master Mix Kit (Qiagen, USA) was performed under the following conditions: 94 °C for 3 min, followed by 28 cycles of 94 °C for 30 s, 53 °C for 40 s and 72 °C for 1 min, after which a final elongation step at 72 °C for 5 min was performed. After amplification, PCR products were checked in 2% agarose gel to determine the success of amplification and the relative intensity of bands. Multiple samples are pooled together (e.g., 100 samples) in equal proportions based on their molecular weight and DNA concentrations. Pooled samples were purified using calibrated Ampure XP beads. Then the pooled and purified PCR product was used to prepare DNA library by following Illumina TruSeq DNA library preparation protocol. Sequencing was performed at MR DNA (www.mrdnalab.com, Shallowater, TX, USA) on a MiSeq (Illumina, San Diego, CA) following the manufacturer’s guidelines. Sequence data were processed using a proprietary analysis pipeline (MR DNA, Shallowater, TX, USA). In summary, sequences were depleted of barcodes then sequences <150 bp removed, sequences with ambiguous base calls removed. Sequences were denoised, OTUs generated and chimeras removed. Operational taxonomic units (OTUs) were defined by clustering at 3% divergence (97% similarity). Final OTUs were taxonomically classified using BLASTn against a curated GreenGenes database^43^. Within community diversity (α-diversity) was calculated using Quantitative Insights Into Microbial Ecology (QIIME) software package^44^. Analysis of α-diversity (Shannon index) was performed by a one-way ANOVA. β-diversity was measured by calculating the weighted UniFrac distances^45^ using QIIME default scripts, and weighted UniFrac PCoA biplot was visualized using *EMPeror*^46^.

### Identification of pomegranate metabolites by high performance liquid chromatography and mass spectrometry

All solvents were HPLC grade from Fisher Scientific. Ellagic, formic and phosphoric acid were purchased from Sigma-Aldrich (St Louis, MO). Pure punicalagin A/B was purchased from ChromaDex (Irvine, CA) and urolithin A was purchased from Jinan Feiteng Technology (Jinan Shandon, China). The composition of the pomegranate extract was analyzed by HPLC and diode array detection. To determine the concentration of urolithin A glucuronide and dimethylellagic acid glucuronide in urine, samples (1 mL) were diluted with 1 ml of 2% formic acid MeOH, vortexed for 30 s and centrifuged at 20,000xg for 10 min at 4 °C. The supernatant was analyzed by LC-MS/MS^8^. The concentration was estimated based on urolithin A standard. The conversion of urolithin A glucuronide to urolithin was estimated by using β-glucuronidase to catalyze hydrolysis of β-D-glucuronic acid residues from urolithin A glucuronide in human urine samples.

### Statistical analysis

To obtain an estimate of the power of this study to detect a treatment effect for the primary outcome we use data from Kasai *et al*.^17^. On the basis of this data we estimated power for this study assuming a treatment effect of similar magnitude and we use a two sample t-test. Based on these assumptions, a final sample size of 20 per group will have 85% power to detect a difference in mean over time among the groups with a 0.050 two sided significance level. We assume that there will be a 20% drop out rate and the goal was to randomize 24 subjects per group. The study was stopped when at least 24 subjects for each group completed the intervention.

Summary statistics (mean, standard deviation and frequency distribution) were generated for baseline demographic and clinical information for each study group to characterize the study population. ANOVA (analysis of variance) and Chi-square test were used to evaluate the difference between treatment groups for continuous variable and categorical variables, respectively. The 12 week outcomes were compared between study groups, using ANCOVA (analysis of covariance) with the adjustment of baseline values. Data management, variable transformations, and other statistical analyses were conducted using SAS 9.2 (Statistical Analysis System, Cary, NC, 2008). Difference in changes of bacterial relative abundances over 12 week intervention among the treatment groups were compared using the non-parametric Kruskal–Wallis test in IBM SPSS Statistics version 23. Only the significant genera and species from the Kruskal–Wallis test were further tested with Mann–Whitney test to assess the differences between treatments. Bonferroni correction was used to correct the probability for multiple comparisons. *P* values < 0.05 were considered statistically significant.

## Data Availability

The datasets generated during the current study are available from the corresponding author on reasonable request.
